# Occupational Paraquat and Glyphosate Exposure May Decline Renal Functions among Rural Farming Communities in Sri Lanka

**DOI:** 10.3390/ijerph18063278

**Published:** 2021-03-22

**Authors:** K.S.M. Abdul, P. Mangala C.S. De Silva, E.M.D.V. Ekanayake, W.A.K.G. Thakshila, S.D. Gunarathna, T.D.K.S.C. Gunasekara, S.S. Jayasinghe, H.B. Asanthi, E.P.S. Chandana, G.G.T. Chaminda, S.H. Siribaddana, Nishad Jayasundara

**Affiliations:** 1 Institute of Biomedical and Pharmaceutical Sciences, Guangdong University of Technology, Guangzhou 510006, China; khajashameem@gdut.edu.cn; 2 Department of Zoology, Faculty of Science, University of Ruhuna, Matara 81000, Sri Lanka; dilini@zoo.ruh.ac.lk (E.M.D.V.E.); thakshilawanniarachchi@gmail.com (W.A.K.G.T.); sakuntha@zoo.ruh.ac.lk (S.D.G.); gunasekara.sc166@fgs.ruh.ac.lk (T.D.K.S.C.G.); 3 Department of Biomedical Sciences, School of Medicine and Health Sciences, University of North Dakota, Grand Forks, ND 58203, USA; 4 Department of Pharmacology, Faculty of Medicine, University of Ruhuna, Galle 80000, Sri Lanka; sudheerasj@med.ruh.ac.lk; 5 Department of Limnology, Faculty of Fisheries and Marine Sciences and Technology, University of Ruhuna, Matara 81000, Sri Lanka; asanthi@fish.ruh.ac.lk; 6 Department of Biosystems Technology, Faculty of Technology, University of Ruhuna, Matara 81000, Sri Lanka; epschandana@zoo.ruh.ac.lk; 7 Department of Civil Engineering, Faculty of Engineering, University of Ruhuna, Hapugala 80000, Sri Lanka; tusharac@cee.ruh.ac.lk; 8 Department of Medicine, Faculty of Medical & Allied Sciences, Rajarata University, Saliyapura 50008, Sri Lanka; sisira.siribaddana@gmail.com; 9 The Nicholas School of the Environment, Duke University, Durham, NC 27708, USA; nishad.jayasundara@duke.edu

**Keywords:** herbicides, glyphosate, biomarkers, farmers, chronic kidney disease, CINAC, Sri Lanka

## Abstract

Extensive use of herbicides is common among rural agricultural workers in Sri Lanka. Recent studies have postulated their role in the development of chronic kidney disease of unknown etiology (CKDu). Paraquat and glyphosate are leading herbicides used by sugarcane farmers (SF), hence occupational exposure is inevitable. This study examined the expression of urinary paraquat, glyphosate and biomarkers among residential SF in CKDu emerging regions, Warunagama (WA) and Rahathangama (RH), in the Uva Province with non-endemic Matara (MA) in the Southern Province of Sri Lanka. Urinary glyphosate, Paraquat, kidney injury molecule -1 (KIM-1), neutrophil gelatinase-associated lipocalin (NGAL) and β2-microglobulin (B2M) were determined using enzyme-linked immunosorbent assays (ELISA). Urinary creatinine, microalbumin, serum creatinine (SCr), serum cystatin C, estimated glomerular filtration rate (eGFR), and albumin creatinine ratio (ACR) were also assessed. Generally, herbicide residues and kidney injury biomarkers were higher in SF compared to the non-endemic MA. Creatinine-adjusted urinary glyphosate and paraquat levels were significantly higher in WA compared to MA. ACR in RH (median 14.9; IQR 5.4–393.1 mg/g) and WA (23.7; 11.5–64.6) was significantly higher than MA (4.3; 2.2–6.7). This study reports 39 individuals with impaired kidney function among SF in Sri Lanka for the first time. Urinary NGAL levels were significantly higher in both WA (median 2.14; IQR 1.28–6.15 ng/mg Cr) and RH (3.09; 1.15–9.09) compared to MA (1.28; 0.56–2.81). However, urinary KIM-1 levels in RH (3.2; 1.29–106.1 ng/g Cr) and WA (3.6; 1.94–115.1) were not significantly higher in MA (1.74; 0.76–116.9). Urinary NGAL (r = 0.493), eGFR (r = −0.147) and ACR (r = 0.171) significantly correlated with urinary glyphosate, but not with urinary paraquat levels. Urinary KIM-1 levels did not correlate with either urinary glyphosate or paraquat, while urinary B2M and serum cystatin C levels showed significant correlation with urinary glyphosate levels. The current study reports higher urinary herbicide levels among sugarcane farmers in WA and RH, and that is potentially linked to the subsequent decline in kidney function, as indicated by ACR, eGFR, and NGAL. We posit that these indicators may serve as markers to detect renal injury among herbicide-exposed SF in Rural Sri Lanka.

## 1. Introduction

Herbicide use is increasing in the world in a quest to scale up crop production [1]. In Sri Lanka, rice production is grown by 1.8 million tons, contributing to an annual production of 2.7 million tons. Weeds cause 30%–40% loss in the rice yield [2]. Historically, weed control was mainly done manually, but labor migration and increased labor costs have led to the increased use of herbicides. Paddy, sugarcane, and vegetable farmers (90%) use herbicides to control weeds in Sri Lanka [2]. Glyphosate (N-(phosphonomethyl) glycine, C_3_H_8_NO_5_P) is one of the commonly used herbicide in Sri Lanka. Since the 1990s it has been commercially available in a variety of formulations. Glyphosate is released into the environment mostly due to reckless mixing, application, and cleaning of sprayers [3]; resulting in contaminated food and water that serve as the main routes of uptake in humans [4]. Glyphosate exposure may contribute to the development of a wide variety of chronic diseases such as developmental disorders (autism), allergies, gastrointestinal diseases, endocrine dysfunction, cardiovascular diseases, Alzheimer’s disease, Parkinson’s disease, cancer and infertility [5,6,7,8]. Paraquat (1, 1 dimethyl–4, 4′–bipyridium dichloride) is a non-selective contact bipyridilium herbicide in frequent use since the 1980s [9]. Farmers are often exposed by occupational, homicidal, or accidental exposure [10,11,12,13,14]. Paraquat may enter the human body through various routes such as oral, nasal, ocular and dermal routes [15,16,17,18]. Paraquat accumulation has been observed in lungs and kidneys, and more likely to affect humans internally [12,19]. Paraquat also exerts its effects externally on the skin and eyes.

Chronic kidney disease of unknown etiology (CKDu) also termed chronic interstitial nephritis in agricultural communities (CINAC) is a rapidly growing public health concern in Sri Lanka [20]. CINAC is mainly seen in North Central Province with scattered pockets in Uva and North Western Provinces [21,22]. A similar disease is prevalent among sugarcane workers in low lands of Nicaragua, El Salvador, and Honduras (referred to as Meso American Nephropathy-MEN), coconut, sugar, and rice farmers in India (referred to as Uddanam nephropathy) and some other parts of South East Asia and Egypt [23,24,25]. Multiple causes have been suggested such as chronic low dose exposure to multiple heavy metals [26] and agrochemicals [27], heat stress and recurrent dehydration [28,29,30,31] nephrotoxic drugs [32], hyperuricemia and hyperuricosuria [29], leptospirosis [33], and genetic susceptibility [34]. Heat stress and associated dehydration were recently highlighted as main etiological factors mainly in MEN [35,36,37,38].

Recently we have shown that heat stress alone cannot cause CINAC [39]. Instead, toxic origin has been highlighted in CINAC and it is postulated that simultaneous exposure to multiple heavy metals and pesticides in the presence of water hardness could damage kidneys in farmers [27,40]. This hypothesis is also supported by recent studies that have found unacceptable levels of agrochemicals particularly glyphosate, its metabolites, 2.4-D, Chlorpyrifos, naphthalene derivatives, and heavy metals of anthropogenic origin in rural agricultural farmers in CINAC hot spots [40,41].

According to KDIGO (Kidney Disease Improving Global Outcomes) clinical practice guidelines, the incidence of structural and functional renal abnormalities persistent for more than three months is defined as CKD. Clinically, reduction of glomerular filtration rate (GFR) to <60 mL/min/1.73 m^2^ or increase in albuminuria (albumin excretion rate; AER ≥30 mg/24 h and albumin to creatinine ratio; ACR ≥30 mg/g (≥3 mg/mmol)) are considered as the main functional criteria for diagnosis. CINAC is a more severe type of CKD that is predominantly prevalent among agricultural communities and it cannot be attributed to the conventional etiologies of CKD such as type 2 diabetes mellitus, hypertension, or glomerular diseases. The exact etiology remains still unknown. Clinically, eGFR is a measure of clearance of wastes in circulation by kidneys and it is treated as the best indicator of overall kidney function and performance. In addition, serum creatinine (SCr), blood urea nitrogen (BUN), serum cystatin C and ACR are commonly used in diagnosis [42]. However, these diagnostic tools may not be sensitive enough to detect early renal damage [43,44]. Several markers predominantly expressed in the proximal tubule, including Kidney Injury Molecule (KIM-1), Neutrophil Gelatinase-Associated Lipocalin (NGAL) and β2-microglobulin (B2M) have been identified as potential indicators of tubular damage [45,46,47]. KIM-1 is a type 1 membrane protein consisting of two domains (i.e., immunoglobulin and mucin), up-regulated in renal proximal tubule upon injury [42,48,49]. NGAL is an iron transporting protein and reported to be expressed in kidney tubules and urine due to acute kidney injury or renal toxicity [50,51]. It is relatively small, resistant to protease activity, and readily available in urine for detection [46]. B2M is a low molecular weight protein that is found in almost all nucleated cells and most biological fluids, including urine and serum. Elevated excretion of B2M is observed as an early indicator of renal injury prior to detectable changes in SCr [52].

Urinary KIM-1, NGAL and B2M are known indicators of renal injury prior to detectable changes in serum creatinine and serve as potential biomarkers in predicting tubulointerstitial damage [45,48,53]. Recently, KIM-1 and NGAL have been suggested to be useful biomarkers for determining renal injury in rural Sri Lankan paddy farmers [54]. However, the association of frequently used herbicides with renal outcomes has not been adequately studied among rural sugarcane farmers in Sri Lanka. Therefore, the objective of this study was to assess glyphosate and paraquat levels in urine and associated renal damage among the rural farmers in Sri Lanka, utilizing conventional and emerging novel biomarkers.

## 2. Materials and Methods

### 2.1. Study Locations and Populations

**Figure 1:**
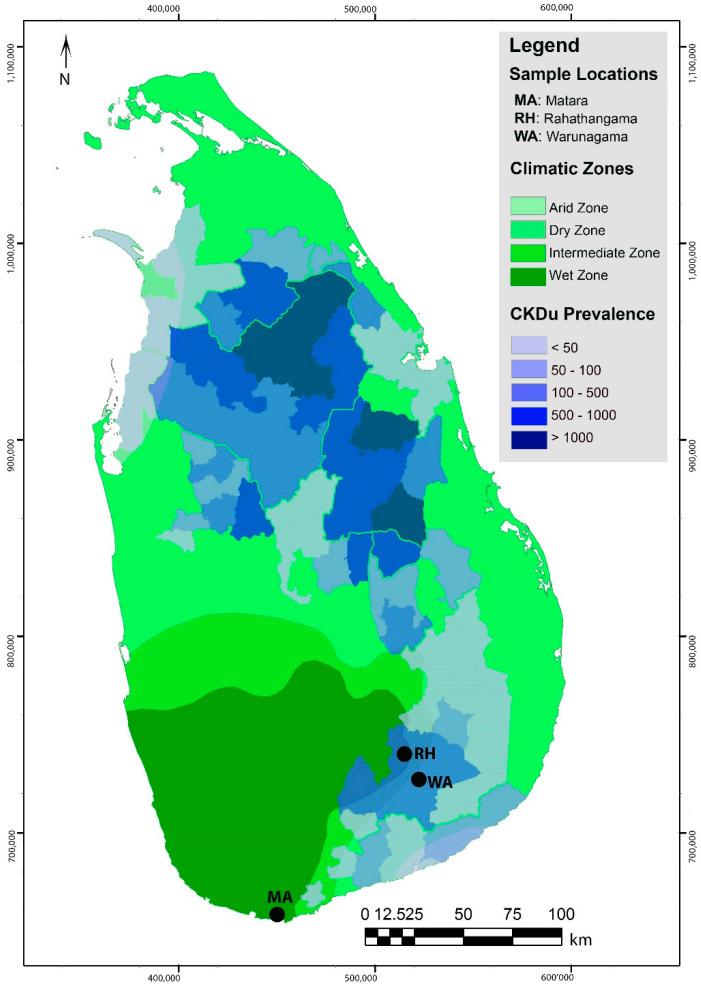
Study locations with respect to climatic zones and CKDu prevalence in Sri Lanka. CKDu prevalence is reported as the number of reported cases at the Divisional Secretariat level [55].

The present study was carried out at three locations in Rahathangama (RH) and Warunagama (WA) Grama Niladhari Divisions (GND) in the Uva province, Kokawela and Kekanadura GNDs in Matara (MA) in the Southern Province of Sri Lanka. Both RH and WA are 3–5 km away from the largest sugarcane mill in Sri Lanka. Sugarcane farming is predominant in RH and WA, which are in the dry zone of the country, whereas paddy and vegetables are predominant in the wet zone MA (Figure 1).

**Figure 2:**
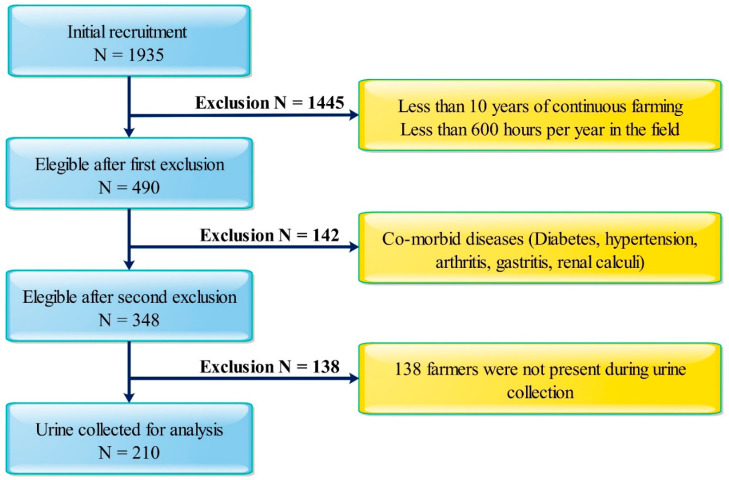
Flow chart representing study population and study design in CKDu emerging locations (RH and WA) and non-endemic location (MA).

Farmers over 20 years of age (*n* = 1935) representing RH, WA and MA were initially recruited based on the electoral list of the locations. An interviewer-administered, pretested survey questionnaire was used to collect demographic data. Our objective was to select a probable highly herbicide exposed group. Therefore, participants (*n* = 1445) with less than 10 years of farming and lower working hours (<600 h per year) were first excluded. During the interview, selected farmers (*n* = 490) were also screened for co-morbidities based on previous medical history (i.e., diabetes, hypertension, arthritis, gastritis, renal calculi, CKD, etc.), and 142 were excluded. Finally, 348 farmers from all three locations were selected but 138 did not turn up for the sample collection. Details of the screened farming populations are illustrated in Figure 2.

### 2.2. Sample Collection

The study was conducted as a community screening session in three areas with emerging evidence of the incidence of CKDu from 2017 to 2018. An initial awareness session was conducted for the participants and sterile containers (50 mL) were delivered to the participants along with instructions for sample collection. The first void morning urine sample was obtained from each participant between 6.00 am to 8.00 am. A preliminary dipstick screening for proteinuria was performed onsite with Combina 13 IVD test strips (Human GmbH, Wiesbaden, Germany) and an automated dipstick reader (Combilyzer [13], Human GmbH, Germany). A non-fasting blood sample (5 mL) was collected from each individual into a sterile serum separator tube. The samples were centrifuged at 3500 rpm for 20 min at 37 °C and isolated serum was transferred into plain vacutainer tubes. The urine and serum samples were temporarily stored at 2–4 °C during transit to the Clinical laboratory of the Department of Zoology, Faculty of Science, University of Ruhuna, Sri Lanka. Immediate assessment of creatinine in serum samples and albumin and creatinine in urine samples was performed. Portions of urine samples were stored at −80 °C for subsequent ELISA assays.

### 2.3. Determination of SCr, UCr and eGFR

Creatinine was measured by modified kinetic Jaffe reaction to minimize interference of non-creatinine and Jaffe-positive compounds in Dimension^®^ clinical chemistry system (Siemens, New York, NY, USA). Picrate reacts with creatinine to produce a red chromophore in the presence of a strong base (NaOH). Absorbance was measured at 510 nm (assay range: 0–20 mg/dL). SCr and UCr were expressed in mg/dL. Estimated glomerular filtration rate (eGFR) was calculated using CKD-EPI (CKD Epidemiology Collaboration) creatinine equation [56].

### 2.4. Determination of Urinary Microalbumin

Urinary microalbumin (UMb) was measured based on particle-enhanced turbidimetric inhibition immunoassay (PETINIA) using Dimension^®^ clinical chemistry system (Siemens, New York, NY, USA). In the presence of human albumin-bound particle reagent (PR), albumin present in the sample competes for monoclonal antibody (mAb) and reduces the rate of PR—mAb aggregation. Therefore, the rate of aggregation is inversely proportional to albumin concentration in urine samples. The rate of aggregation was measured using bichromatic turbidimetric reading at 340 nm (assay range: 1.3 °C 100 mg/L). UMb level was later used to calculate ACR and participants with higher ACR (≥30 mg/g Cr) were retested after 3 months of initial collection. Ion-exchange high-performance liquid chromatography (HPLC) was used for the measurement of HbA1c using Bio-Rad D-10^TM^.

### 2.5. Determination of Urinary Glyphosate and Paraquat

Glyphosate ELISA kits (Cat. No: EL0054-003) and Paraquat ELISA kits (Cat. No: EL0054-002) were obtained from US Biocontract Inc., San Diego, CA, USA. Before the start, all reagents, kits, standards and urine samples were brought to room temperature. Urine samples were centrifuged at 1500 rpm for 10 min and the supernatants were used for the assay. The assay procedure of the competitive ELISA was followed according to the manufacturer’s instructions. Absorbance was measured at 450 nm using a microplate reader (Utrao microplate reader—SM600, Shanghai Yong Chuang, China). Glyphosate and Paraquat concentrations in urine were determined using a 4PL nonlinear regression model and adjustment for creatinine was done accordingly.

### 2.6. Measurement of Renal Injury Biomarkers

Analysis of kidney injury biomarkers; KIM-1, NGAL, beta 2 microglobulin (B2M) in urine samples and Cystatin C (Cys C) in serum samples was done using in vitro Enzyme-Linked Immunosorbent Assay (ELISA) kits according to the assay protocol of the manufacturer. For the assays, KIM-1 (detection range: 0.312–20 ng/mL; sensitivity: <0.043 ng/mL), B2M (detection range: 0.03–10 μg/mL; sensitivity: 0.039 μg/mL), Cys C (detection range: 7.8–500 ng/mL; sensitivity: 5.824 ng/mL), ELISA kits (CUSABIO Technology LLC, China) were used. Intra-assay precision (CV%) was <8% and inter-assay precision (CV%) was <10%. For the assessment of NGAL, ELISA kits (detection range: 4–1000 pg/mL; sensitivity: <4 pg/mL; Ray Biotech, Inc., Norcross, GA, USA) were used. Intra-assay precision (CV%) was <10%) and inter-assay precision (CV%) was <12%. Absorbance was measured at 450 nm with correction wavelength at 570 nm using microplate reader (Utrao microplate reader—SM600, Shanghai Yong Chuang, China). Biomarker concentrations of the samples were determined with a 4 PL regression model.

### 2.7. Statistical Analysis

Data were analyzed using SPSS Statistics (version 22.0; IBM Inc., New York, NY, USA). Continuous variables were reported as mean (SD) or median (IQR) whereas categorical variables were reported as proportions. Characteristics of participants between locations were assessed by 2 sided χ2 test. Glyphosate and paraquat levels and renal biomarkers were adjusted for urine creatinine concentrations. Comparisons of renal biomarkers between geographical locations were performed by one-way ANOVA test with normally distributed parameters or transformed to natural log parameters. Kruskal–Wallis test and Mann–Whitney test with Bonferroni adjustment were performed when deviated from the normality. Multiple linear regression was used to examine the associations of renal biomarkers with age, location, gender, and urinary glyphosate and paraquat residues.

### 2.8. Ethics Statement

The ethics review committee of the Faculty of Medicine, University of Ruhuna, Matara, Sri Lanka approved (Ref. No: 09.03.2016:3.2) the study. A written or a thumbprint consent was obtained from each participant and the study was conducted according to the Helsinki declaration.

## 3. Results

Baseline characteristics and lifestyle of farmers from RH (*n* = 69), WA (*n* = 66) and MA (*n* = 75) are given in Table 1. Face masks/respirators, protective clothing, gloves, goggles, and boots were identified as the personal protective equipment (PPE) among the farmers. The percentage of PPE use was calculated based on the essential use of a face mask/respirator along with at least one of the other types of PPE mentioned above.

Smoking and alcohol consumption were highest in WA followed by RH and MA. In the past, residents in RH and WA were mostly dependent on surface wells for drinking water. However, presently WA participants were actively using tap water supplied by the government. Recently, very few participants from RH shifted towards tap water consumption but still, many participants are using surface well water for their daily consumption. Participants from MA had access to tap water in the past and the present. Farmers from RH and WA cultivate mostly sugarcane and some vegetables whereas no farmers in MA cultivate sugarcane but paddy and vegetables. The active use of herbicides and fertilizers was predominant in all three study locations. However, the intensive use of herbicides was reported in RH and WA. Moreover, more than half of the participants in each location involved in mixing different herbicide formulations without proper understanding. Most notably, they received recommendations for herbicides from unauthorized sellers ignoring the agricultural officers. Overuse of herbicides was common among participants from all three study locations. Less than 40% of all participants preferred using safety measures while handling/applying herbicides. No proper storage methods were used by the farmers. They were unaware of the correct disposal of empty containers and even preferred to reuse the empty containers.

### 3.1. Exposure Assessment of Urinary Glyphosate and Paraquat

Detectable levels of urinary glyphosate and paraquat were observed in all participants from the three study locations (Table 2). Creatinine-adjusted urinary glyphosate and paraquat were higher in WA and RH than MA. Creatinine-adjusted urinary glyphosate levels were significantly higher in WA compared to MA. Female farmers had higher creatinine adjusted urinary glyphosate levels compared to male farmers in all three locations. In general, creatinine-adjusted urinary paraquat levels were significantly higher in WA farmers compared to MA farmers. However, no statistical difference was found between RH and MA farmers. Male farmers in WA reported the highest urinary paraquat followed by RH and MA. Creatinine-adjusted urinary paraquat levels were significantly higher in males from WA and RH with compared to the males in MA (Table 2).

### 3.2. Assessment of Renal Biomarkers

**Figure 3:**
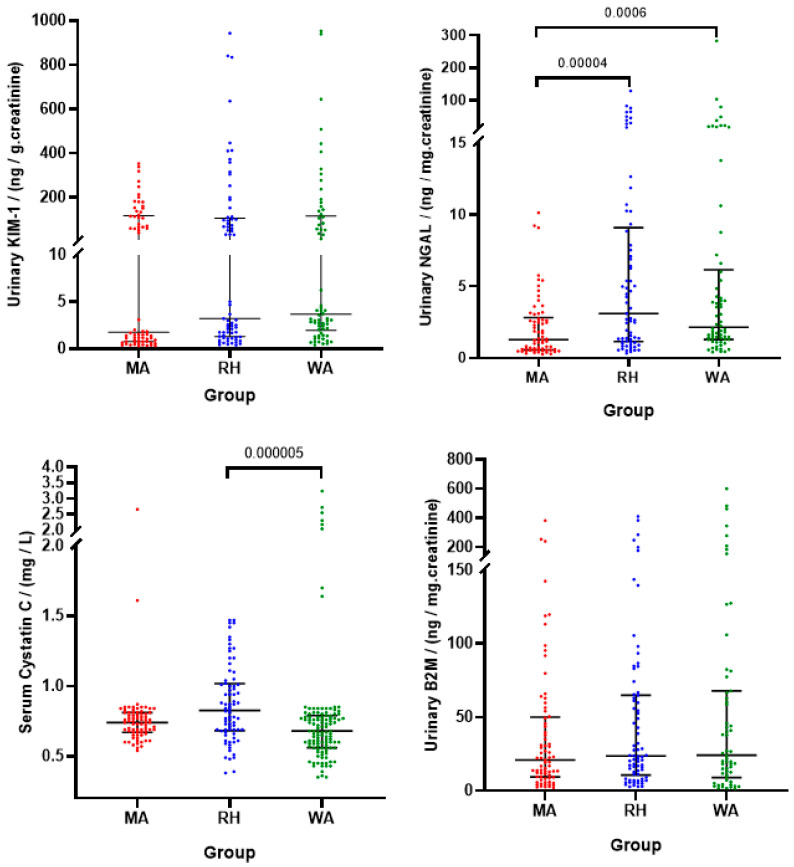
Renal injury biomarker levels (median and IQR) in emerging locations (Rahathangama (RH) and Warunagama (WA)) and non-endemic Matara (MA).

Kidney function as evaluated in terms of microalbumin, creatinine, ACR, SCr, eGFR, and the expression of renal biomarkers, KIM-1, NGAL, B2M, and Cys C are given in Table 3 and further illustrated in Figure 3.

Urinary microalbumin was higher in both male and female farmers in sugarcane farming locations (RH & WA) compared to MA, however urinary creatinine in MA was higher than in WA and RA. In general, Albumin to creatinine ratio (ACR) in RA and WA farmers was significantly higher than MA farmers. However, no statistical significance was reported between the two sugarcane farming locations, RH and WA. The highest ACR was observed in males from RH and WA followed by MA. Moreover, females from RH and WA also had higher ACR levels compared to the females from MA. Albuminuria (ACR ≥ 30 mg/g) and eGFR (eGFR < 60 (mL/min/1.73 m^2^) in repeated occasions were reported in all three farming locations and defined as CKDu cases under WHO-SL study case definition [41]. The highest number of CKDu cases were reported in WA (14 men and 10 women), followed by RH (11 men and four women) and only three males were reported from MA. The highest SCr was reported in WA followed by RH and MA. SCr in WA was significantly higher compared to MA farmers but not with RH farmers. Moreover, eGFR values of farmers in both sugarcane farming locations were significantly lower than the MA farmers.

### 3.3. Assessment of Kidney Injury Using Urinary Biomarkers

Table 3 indicates differences in renal biomarkers as indicators of kidney injury. Creatinine-adjusted urinary KIM-1 levels (mean ± SEM) in MA, RH and WA were 34.9 ng/g Cr, 114.6 ng/g Cr and 101.6 ng/g Cr respectively. KIM-1 expression in RH and WA was approximately two times higher than MA, but creatinine-adjusted urinary KIM-1 levels in both RH and WA were not significant compared to KIM-1 levels in MA. Creatinine-adjusted urinary NGAL levels in RH (12.0 ng/mg Cr, mean ± SEM) and WA (12.4 ng/mg Cr, mean ± SEM) was six times higher than the NGAL levels in MA farmers (2.1 ng/g Cr, mean ± SEM). Urinary NGAL levels were significantly higher in RH and WA compared to MA. Further, urinary B2M levels showed no significant difference among the locations while serum Cys C levels in RH farmers (0.87 mg/L, mean ± SEM) were significantly higher than that of WA farmers (0.77 mg/L, mean ± SEM).

### 3.4. Association between Herbicide Exposure and Kidney Functions

Associations between ACR, SCr, eGFR, KIM-1, NGAL, B2M, and Cys C with glyphosate and paraquat residues are indicated in Table 4.

Creatinine-adjusted urinary glyphosate significantly correlated with ACR (r_s_ = 0.171; *p* = 0.015). Furthermore, creatinine-adjusted urinary glyphosate significantly correlated with eGFR (r_s_ = −0.147, P = 0.036) and NGAL (r_s_ = 0.4932, *p* = 0.001). No correlation between glyphosate with Kim−1 and SCr was found. A positive correlation between ACR and eGFR with creatinine-adjusted urinary paraquat levels was also observed, but the association was not significant. Further, creatinine-adjusted urinary B2M levels and serum Cys C levels were significantly correlated with urinary glyphosate levels, but not with urinary paraquat levels. Based on a multiple linear regression analysis, age and gender significantly predicted changes in the renal biomarkers between locations. Further, urinary glyphosate levels significantly correlated with the expression of urinary NGAL in all three farming locations. Overall, SCr, eGFR, ACR, NGAL, and Cys C significantly correlated with the location indicating the presence of some region-specific factors that are capable of affecting renal function (Table 5).

## 4. Discussion

Farmers from all three study locations were found to have glyphosate and paraquat in urine, indicating previous exposure and was supported by the information collected in performed pesticide survey. Pesticide excretion was higher in both sugarcane-farming locations (WA and RH) when compared to the location where paddy and vegetables are cultivated (MA). Higher herbicide residues reaffirm that exposure intensity may be higher among SF. Occupational exposure to herbicides has been previously reported by many studies [11,12,27,57,58,59,60] and may occur probably through dermal contact of herbicides [10,17,61]. Moreover, inhalation of aerial spraying particles and oral intake during mixing may also lead to further systemic exposure [13]. Inadequate safety measures and lack of personnel protective equipment were common among the farmers hence exposure through skin and the respiratory tract was inevitable. In a previous study, avoiding safety measures during preparation and application of glyphosate was associated with higher glyphosate residues in farmers [57].

Glyphosate in urine may reflect the exposure and used in bio-monitoring [57,62,63,64]. This study reports higher urinary glyphosate among sugarcane farmers in RH and WA compared to the MA farmers. Previous studies have also found urinary glyphosate among rural farmers in Sri Lanka. Moreover, detectable levels of glyphosate (0.075–3.36 µg/L) and its metabolite AMPA (0.075–2.65 µg/L) were found in CKDu patients [40,41]. Further, glyphosate residues were found in maternal serum collected from Thai women [60]. In this Thai study, the occupation has been considered as the main risk factor while living near an agricultural field was also found as a co-factor. They also found glyphosate residues in people engaged in other occupations highlighting possible dietary exposure. Moreover, a recent study reported that glyphosate levels in urine may be due to consumption of conventional food over organic food [63]. Therefore, multiple routes of exposure remain possible for RH and WA farmers.

We also report detectable levels of urinary paraquat among three locations. Dermal contact and inhalation are mainly responsible for urinary paraquat levels [65,66]. Sugarcane farmers in Sri Lanka predominantly used paraquat in the 1980s and paraquat spraying was only done by the male farmers. This may explain the detectable paraquat in male farmers in this study. Paraquat was previously observed among spray handlers [12] and moderate to high dermal exposure was reported among paddy farmers in Malaysia [65]. However, study in Thailand revealed the presence of in the serum of pregnant women who involved in land preparation and agricultural work during the last trimester of pregnancy. [60]. Apart from renal outcomes, paraquat exhibits its toxicity primarily on the respiratory system. Acute exposure to paraquat might become fatal due to respiratory failure accompanied by oxidative damage to the alveolar epithelium with subsequent pulmonary fibrosis [67]. However, paraquat has been shown to exert significant toxic effects on several organs including kidneys [68]. Particularly, renal damage mediated by paraquat exposure has been observed in diverse farming communities and the renal damage is reflected through urinary excretion of renal injury biomarkers [69,70,71].

This is the first study that reports 39 cases with high ACR from sugarcane farming locations in Sri Lanka. Co-morbid diseases (i.e., diabetes, hypertension, pyelonephritis, renal calculi, etc.) that may influence levels of urinary ACR were excluded. Therefore, reported 39 cases can be confirmed as new CKDu cases from the Sugarcane farmers. CKDu is highly prevalent in North Central Province (NCP) of Sri Lanka, especially among paddy farming communities [20,21]. However, this study confirms that CKDu is not restricted to paddy farmers, but also prevalent among sugarcane farmers. A recent study reported a prevalence of 6.9% of micro-albuminuria in Salvadoran farmers [72]. A similar form of decline in kidney function in Nicaraguan sugarcane workers has also been reported leading to the development of CKD [73]. Here, we also report an association between urinary glyphosate and ACR. The decline of kidney functions indicated by elevated ACR could be a consequence of repeated occupational exposure to toxic substances such as herbicides, and further exposure might lead to the development of CKDu.

Biomarkers such as N-acetyl-β-d-glucosamininidase, Cystatin C, Interleukin 18, liver fatty acid-binding protein, Netrin-1, insulin-like growth factor-binding protein 7, tissue inhibitor of metalloproteinases-2 have been used to assess the kidney injury [74]. KIM-1 and NGAL have been used for the characterization of kidney injury among Sri Lankan farmers [54]. Associations between glyphosate with renal markers ACR, eGFR, NGAL have also been noted. This indicates the utility of ACR, eGFR, and NGAL as potential markers for glyphosate-induced kidney injury among farmers. Wunnapuk et al. (2014) [75] reported KIM-1 as an early biomarker of glyphosate-induced injury in a rat model. However, we report no association between KIM-1 and glyphosate, despite reporting higher KIM-1 in sugarcane farmers. Similarly, paraquat may also stimulate KIM-1 and NGAL in acute intoxication [76,77,78]. However, no significant association was found in our study.

The main strength of our study is the exposure assessment of commonly used glyphosate and paraquat among rural sugarcane farmers in Sri Lanka in relation to kidney function. No study has so far been reported reflecting both herbicide exposure and kidney function in CKDu hotspots around the globe. This is also the first study carried in sugarcane farming locations in Sri Lanka that reports the first cases of kidney impairment with an unknown etiology.

The main limitation of this study is the sample size. We applied precise exclusion criteria in two occasions despite the initial recruitment of 1935 farmers from all three farming communities. The exclusion of farmers less than 10 years of farming and less than 600 h in the field enabled us to include highly exposed farming groups. We also eliminated farmers with co-morbid diseases (*n* = 142) to get a reliable estimation of CKDu prevalence among highly exposed farmers. Even with the exclusion criteria, we recruited 348 farmers but the absence of 138 farmers in urine sampling accounted a moderate sample size. However, when assessed on the regional level, some of the parameters were considerably heterogeneous across the three study locations. In multiple regression analysis, SCr, eGFR, ACR, NGAL, and Cys C showed significant correlations with the location. This heterogeneity might arise either due to the small sample size and uneven male: female ratio in the three study locations or due to some other factors of environmental, occupational, lifestyle or biochemical contexts that might impact on renal health of the inhabitants. Further, the three study locations exhibit notable differences in lifestyle and agricultural practices. However, within the context of current findings, it is difficult to conclude whether the observed heterogeneity occurred due to actual population characteristics or due to the small sample size.

CKDu or CINAC is very likely a multifactorial disease and a wide spectrum of potential risk factors have been proposed with scientific evidence [55,79]. The present study merely renders the prevalence of the disease among selected farming communities in Sri Lanka, and provide no deep insight into the etiology of the observed cases is presented. However, alterations in renal function characterized by ACR or tubular proteinuria might be resulted by a variety of pathological conditions including AKI, and acute tubular nephritis. Renal biopsies would be highly important in the characterization of such pathologies although it has not been performed in the present study. Thus, follow-up studies with the communities are recommended to detect any persistent alterations or progressive decline in renal function of the farmers in study areas. Interestingly, creatinine-adjusted mean urinary KIM-1 levels in RH and WA farmers appear to be nearly two-fold higher than that of MA farmers, although there exists no significant difference among the farming groups. This might be due to considerably high variation of individual values and increasing sample size might yield more precise results.

We also used the ELISA method to estimate urinary glyphosate and paraquat, which may be less sensitive compared to the other analytical techniques. However, ELISA was used in recent studies [40,80] and no statistical difference was found between the two methods [81]. The other main limitation is the lack of reference urinary biomarker levels that reflect subclinical damage in Sri Lankan nephropathy which prevents comparison among herbicide exposed and non-exposed farming communities. Despite recent studies [54,82], validation of normal levels of NGAL and KIM-1 in larger populations has not been carried out. Previously, Jayasumana et al. (year) reported glyphosate and heavy metals (Cd, & As) as the main etiological factor in CKDu among farmers [27] and were shown to affect KIM-1 expression in an animal model [26]. Expression of KIM-1 in response to exposure to toxic metals including As [83], Cd [84], and Hg have been noted in human subjects and Cr [18] in a rat model. However, we did not explore the possible association between heavy metals, herbicides and kidney damage simultaneously.

In summary, we report occupational exposure of glyphosate and paraquat among rural farmers in Sri Lanka. Higher creatinine-adjusted urinary glyphosate and paraquat was found among sugarcane farmers compared to the paddy & vegetable farmers. Thirty-nine new CKDu susceptible cases were reported for the first time in sugarcane farming locations. Renal function markers were lower and tubular injury biomarkers urinary KIM-1 and NGAL were higher in sugarcane farmers indicating declining kidney function. Glyphosate was significantly correlated with ACR, eGFR, and NGAL, although no association was reported with paraquat. This further supports that ACR, eGFR and NGAL may serve as better markers to detect kidney injury among glyphosate-exposed farmers in rural Sri Lanka.

It is evident that farmers require better practices when handling pesticides. The use of PPE, particularly respirators, was found very low as a whole, indicating high risk of toxic. Further, excessive use of pesticides above the recommended usage was also common among the farmers. This might lead to a high degree of environmental pollution, food and water contamination, and associated health risks to other community members. Thus, we recommend incentives and awareness programs to improve the safety practices and positive attitudes on pesticide use among the farmers in study areas. Further, continuous monitoring of renal function and follow-up studies are needed to detect any further decline in renal function and CKDu susceptibilities among rural farming communities.

## Figures and Tables

**Table 1:** Baseline characteristics of the male and female farmers in the study.

Characteristics	Total (*n* = 210)	Matara (*n* = 75)	Rahathangama (*n* = 69)	Warunagama (*n* = 66)	*p*-Value
Age (Mean ± SEM)	44.39 ± 0.93	39 ± 1.4	48 ± 1.5	47 ± 1.5	<0.0001
Smoking (%)					
Yes	30.46	25.3	21.74	45.45	0.006
Alcohol consumption (%)					
Yes	33.33	25.3	14.49	62.12	<0.0001
Chewing betel (%)					
Yes	44.76	16.00	60.87	60.61	<0.0001
Drinking water (%)—current					
Surface water	40.49	4.00	75.36	4.55	<0.0001
Drinking water (%)—past					
Surface water	62.86	9.33	92.75	92.43	<0.0001
Intensive Herbicide use (%)					
Yes	53.33	10.66	81.20	72.70	<0.0001
Fertilizer use (%)					
Yes	83.44	70.66	81.20	72.70	0.352
Mixing herbicides on-site (%)					
Yes	54.41	50.66	62.32	50.00	0.250
Source of recommendation (%)					
Agriculture officer	8.55	14.60	4.35	6.06	0.107
Usage at the recommended dose (%)					
Yes	21.91	20.00	28.99	16.67	0.194
Overuse of herbicides (%)					
Yes	54.76	44.00	55.07	66.67	0.038
Use of Personal Protective Equipment (PPE) (%)					
Yes	29.49	30.60	39.13	18.18	0.027
Safe storage of herbicides (%)					
Yes	31.89	29.30	36.23	30.30	0.616
Correct disposal (%)					
Yes	32.84	17.30	36.23	46.97	<0.000

**Table 2:** Creatinine-adjusted urinary Glyphosate and paraquat levels in male and female farmers in Matara, Rahathangama and Warunagama.

Variables	Total	Matara (MA)	Rahathangama (RH)	Warunagama (WA)	Kruskal-Wallis & Mann-Whitney U Test
**Glyphosate (µg/g Cr)**					
All (Median)	198.49	177.8	224.3	224.5	<0.0001 ^1^ MA vs. RH 0.033 ^2^ MA vs. WA 0.017 ^2^ RH vs. WA 0.710
Range	0.0–979.98	99.5–350.1	33.1–827.3	0.0–979.9	
IQR	147.01–286.61	147.1–225	138.3–327.7	147.9–353	
*n*	210	75	69	66	
Male (Median)	175.3	175.2	147.2	200.8	0.07 ^1^ MA vs. RH 0.207 MA vs. WA 0.157 RH vs. WA 0.034 ^2^
Range	33.14–555.4	105–287.3	33.14–474.5	41.74–555.4	
IQR	138.3–250.8	148.5–216.8	95.23–238.4	146.2–328.0	
*n*	124	47	31	46	
Female (Median)	234.1	185.3	299.0	276.9	0.012 ^1^ MA vs. RH 0.002 ^2^ MA vs. WA 0.035 ^2^ RH vs. WA 0.901
Range	0.0–827.3	99.5–350.1	83.78–827.3	0–980	
IQR	163.0–343.1	137.2–235.9	177.9–400.7	161.6–494.1	
*n*	86	28	38	20	
**Paraquat (µg/g Cr)**					
All (median)	0.31	0	0.12	0.75	<0.0001 ^1^ MA vs. RH 0.7341 MA vs. WA < 0.0001 ^2^ RH vs. WA 0.0042 ^2^
Range	0.0–11.42	0.0–1.3	0.0–2.8	0.0–11.4	
IQR	0.0–0.99	0–0.63	0–0.92	0–2.1	
*n*	210	75	69	66	
Male (Median)	0.645	0	0.84	1.675	<0.0001 ^1^ MA vs. RH 0.0006 ^2^ MA vs. WA < 0.0001 ^2^ RH vs. WA 0.019
Range	0.0–11.42	0–1.32	0–2.63	0–11.42	
IQR	0.0–1.57	0–0.63	0–1.52.	0.18–2.73	
*n*	124	47	31	46	
Female (Median)	0.00	0.33	0	0.18	0.182 ^1^ MA vs. RH 0.160 MA vs. WA 0.890 RH vs. WA 0.100
Range	0.0–2.79	0–0.65	0–2.79	0–1.17	
IQR	0.0–0.42	0–0.65	0–0.227	0–0.4925	
*n*	86	28	38	20	

Median, Range, IQR are given. ^1^—Kruskal-Wallis test and ^2^—Mann-Whitney U Test with Bonferroni correction (P = 0.05/3; *p* = 0.017) indicate significant differences between the locations and highlighted with bold letters.

**Table 3:** Creatinine-adjusted biomarkers of kidney injury KIM-1, NGAL, B2M, Cys C and kidney functions among MA, RH and WA farming communities.

Variables	Total *n* = 210	Matara *n* = 75	Rahathangama *n* = 69	Warunagama *n* = 66	Mann-Whitney U Test
Microalbumin (mg/L); *p* < 0.0001 ^1^					
Median	10.00	6	11	21	MA vs. RH <0.0001 ^2^
Range	2.00–311.0	2–311	2–162	2–157	MA vs. WA <0.0001 ^2^
IQR	5.00–25.00	4–10	7–36.5	6–65.5	RH vs. WA 0.3052
Urinary Creatinine (mg/dL); *p* < 0.0001 ^1^					
Median	85.40	136.8	58.9	62.15	MA vs. RH <0.0001 ^2^
Range	0.78–513.10	32–513.1	19.9–355.2	7.8–337.4	MA vs. WA <0.0001 ^2^
IQR	45.65–179.10	87.4–259.3	40.7–108.1	34.6–142	RH vs. WA 0.636
ACR (mg/g Cr); *p* < 0.0001 ^1^					
Median	12.00	4.3	14.9	23.7	MA vs. RH <0.0001 ^2^
Range	1.10–470.00	1.1–237.9	5.4–393.1	7.1–470	MA vs. WA <0.0001 ^2^
IQR	6.57–24.55	2.2–6.7	10.8–24.0	11.5–64.6	RH vs. WA 0.055
Serum Creatinine (mg/dL); *p* = 0.003 ^1^					
Median	1.11	1.09	1.13	1.225	MA vs. RH 0.282
Range	0.67–6.41	0.67–4.07	0.67–5.98	0.67–6.41	MA vs. WA <0.0001 ^2^
IQR	0.96–1.33	0.98–1.13	0.91–1.6	1.01–2.07	RH vs. WA 0.060
eGFR (mL/min/1.73 m^2^) *p* < 0.0001 ^1^					
Median	73.00	86	65	59	MA vs. RH <0.0001 ^2^
Range	9.0–123.0	15–122	11–115	9–123	MA vs. WA <0.0001 ^2^
IQR	51.0–90.0	72–98	41–87	36–87	RH vs. WA 0.566
KIM-1 (ng/g Cr); *p* = 0.156					
Median	3.096	1.74	3.2	3.6	MA vs. RH 0.1207
IQR	1.181–112.41	0.76–116.9	1.29–106.1	1.94–115.1	MA vs. WA 0.0807
Mean (SEM)	94.56 ± 12.62	66.5 ± 11.6	114.6 ± 25.5	101.6 ± 25.0	RH vs. WA 0.7165
Range	0.30–954.0	0.3–353.9	0.4–944.2	0.4–954.3	
NGAL (ng/mg Cr); *p* < 0.0001 ^1^					
Median	2.05	1.28	3.09	2.14	MA vs. RH <0.0001 ^2^
IQR	0.91–5.20	0.56–2.81	1.15–9.09	1.28–6.15	MA vs. WA 0.0006 ^2^
Mean (SEM)	8.89 ± 1.83	2.1 ± 0.3	12.0 ± 2.8	12.4 ± 4.7	RH vs. WA 0.5766
Range	0.27–283.01	0.3–10.1	0.3–128.4	0.4–283.0	
B2M (ng/mg Cr) *p* = 0.7403					
Median	22.54	21.01	23.64	24.18	MA vs. RH 0.852
IQR	9.49–61.63	9.41–50.01	10.79–65.03	9.13–68.04	MA vs. WA 0.90
Mean (SEM)	55.74 ± 6.29	42.27 ± 7.32	55.06 ± 9.26	73.26 ± 16.16	RH vs. WA 0.90
Range	1.54–601.14	2.11–382.4	2.75–411.3	1.54–601.4	
Cys C (mg/L); *p* < 0.0001 ^1^					
Median	0.77	0.74	0.825	0.68	MA vs. RH 0.094
IQR	0.69–0.85	0.67–0.81	0.68–1.02	0.56–0.79	MA vs. WA 0.0504
Mean (SEM)	0.87 ± 0.03	0.77 ± 0.03	0.87 ± 0.03	0.77 ± 0.04	RH vs. WA <0.0001
Range	0.38–3.24	0.54–2.66	0.38–1.47	0.35–3.24	

Median, range and IQR were given for each biomarker. ^1^—Kruskal-Wallis test and ^2^—Mann-Whitney U Test with Bonferroni correction indicates significant differences between the locations and highlighted with bold letters.

**Table 4:** Associations between Microalbumin, ACR, serum creatinine, eGFR, urinary biomarkers KIM-1, NGAL with urinary glyphosate and paraquat residues.

Variables	Glyphosate (µg/g Cr)		Paraquat (µg/g Cr)	
	r_s_	*p*	r_s_	*p*
Microalbumin (mg/L)	−0.224	0.0014	0.133	0.059
KIM-1 (ng/g Cr)	0.098	0.165	−0.057	0.418
NGAL (ng/mg Cr)	0.4932	**0.001**	−0.113	0.107
SCr (mg/L)	0.098	0.162	0.021	0.771
eGFR (mL/min/1.73 m^2^)	−0.147	**0.036**	0.051	0.411
B2M (ng/mg Cr)	−0.1438	**0.0416**	−0.06328	0.3721
SCys C (mg/L)	−0.1411	**0.04567**	0.1258	0.0752

Bold letters indicate significant difference; *p* < 0.05.

**Table 5:** Multiple linear regression analysis for the association of glyphosate, paraquat, age and gender and location with renal biomarkers.

	Variable	Total		Matara (MA)		Rahathangama (RH)		Warunagama (WA)	
		*β* (95% CI)	*p*	*β* (95% CI)	*p*	*β* (95% CI)	*p*	*β* (95% CI)	*p*
SCr	Glyphosate Paraquat Age Gender Location	−0.01 (−0.001 to 0.001) 0.168 (−0.001 to 0.02) 0.21 (0.006 to 0.03) −0.11 (−0.52 to 0.09) 0.239 (0.123 to 0.449)	0.87 0.02 0.002 0.16 0.01	6.2 (−0.003 to 0.003) 0.11 (−0.23 to 0.45) 0.01 (0.001 to 0.02) −0.82 (−0.42 to 0.25)	0.96 0.52 0.05 0.63	7.5 (−0.001 to −0.3) −0.3 (−0.5 to 0.03) 0.000 (−0.02 to 0.02) −0.8 (−1.3 to −0.3)	0.92 0.07 0.98 0.001	0.001 (−0.001 to 0.002) −0.04 (−0.2 to 0.1) 0.02 (−0.006 to 0.04) −0.2 (−1.1 to 0.6)	0.54 0.62 0.14 0.59
eGFR	Glyphosate Paraquat Age Gender Location	−0.01 (0.05 to 0.01) −0.14 (−5.5 to 0.17) −0.16 (−0.65 to −0.05) −0.12 (−16.4 to 1.87) −0.3.4 (−15.44 to −6.03)	0.21 0.07 0.02 0.12 <0.001	0.01 (−0.09 to 0.09) −1.92 (−12.9 to 9.1) −1.09 (−1.5 to −0.7) −2.46 (−13.5 to 8.4)	0.98 0.73 <0.001 0.65	−0.02 (−0.07 to 0.02) 7.7 (−1.3 to 16.7) −0.5 (−1.02 to −0.05) 9.7 (−5.6 to 25)	0.26 0.09 0.03 0.21	−0.02 (−6.06 to 0.02) 2.0 (−1.5 to 5.6) −0.9 (−1.4 to −0.3) −27.4 (−39.2 to −3.6)	0.42 0.25 0.002 0.02
KIM−1	Glyphosate Paraquat Age Gender Location	−0.04 (−0.23 to 0.14) 0.05 (−12.61 to 23.85) 0.11 (−0.48 to 3.37) −0.01 (−62.78 to 54.02) 0.08 (−13.15 to 48.24)	0.65 0.54 0.14 0.88 0.261	−0.32 (−0.7 to 0.09) −8.26 (−59.5 to 43) −3.14 (−5.05 to −1.23) −72.04 (−122.8 to −21.3)	0.12 0.75 0.002 0.006	0.03 (−0.3 to 0.4) −33.5 (−106.9 to 39.9) 2.4 (−1.6 to 6.4) 46.5 (−78.9 to 171.8)	0.86 0.36 0.23 0.46	0.05 (−0.24 to 0.34) −10.0 (−35.7 to 15.7) 0.01 (−3.9 to 3.9) −154.3 (−283.9 to −24.8)	0.74 0.44 0.99 0.02
NGAL	Glyphosate Paraquat Age Gender Location	0.09 (−0.01 to 0.04) −0.008 (−2.76 to 2.48) −0.02 (−0.32 to 0.24) 0.12 (−1.97 to 14.82) 0.16 (0.73 to 9.54)	0.23 0.92 0.78 0.13 0.023	0.02 (0.01 to 0.03) −1.14 (−2.3 to 0.01) 0.03 (−0.01 to 0.07) −0.25 (−1.4 to 0.9)	<0.001 0.05 0.19 0.67	0.06 (0.03 to 0.1) −0.3 (−7.8 to 7.2) −0.3 (−0.7 to 0.1) −5.6 (−18.4 to 7.2)	0.001 0.94 0.17 0.39	0.1 (0.07 to 0.2) −3.3 (−7.4 to 0.8) −0.4 (−0.9 to 0.3) −2.2 (−22.7 to 18.3)	<0.001 0.11 0.27 0.83
ACR	Glyphosate Paraquat Age Gender Location	−0.06 (−0.00 to 0.05) 0.17 (1.35 to 17.14) 0.24 (0.57 to 2.24) 0.003 (−24.73 to 25.86) 0.261 (12.45 to 39.11)	0.39 0.02 0.001 0.97 <0.001	0.03 (−0.1 to 0.17) 9.21 (−8.1 to 26.6) 0.50 (−0.14 to 1.15) −2.89 (−20.0 to 14.3)	0.64 0.29 0.12 0.74	−0.02 (−0.2 to 0.1) −30.6 (−61.3 to 0.1) −0.5 (−2.2 to 1.2) −54.6 (−1.7 to −2.1)	0.79 0.05 0.54 0.04	0.02 (−0.1 to 0.2) 1.7 (−10.5 to 13.9) 1.6 (−0.3 to 3.4) −4.6 (−66.3 to 56.9)	0.82 0.78 0.1 0.88
B2M	Glyphosate Paraquat Age Gender Location	−0.03 (−0.17 to 0.12) 0.02 (−12.33 to 16.22) 0.07 (−0.75 to 2.27) −0.09 (−70.9 to 20.56) −0.013 (−24.00 to 21.88)	0.75 0.79 0.32 0.28 0.854	−0.07 (−1.22 to 0.69) −0.11 (−187.55 to 74.28) −0.10 (−5.95 to 2.77) −0.14 (−175.43 to 53.08)	0.58 0.39 0.47 0.29	−0.02 (−0.15 to 0.13) −0.22 (−50.98 to 4.74) 0.08 (−1.00 to 2.03) −0.24 (−85.68 to 9.48)	0.89 0.10 0.50 0.12	−0.09 (−0.22 to 0.11) 0.23 (−2.43 to 27.02) 0.33 (0.87 to 5.38) 0.07 (−56.89 to 91.64)	0.53 0.1 0.007 0.64
Cys C	Glyphosate Paraquat Age Gender Location	−0.001 (0.00 to 0.00) 0.09 (0.02 to 0.06) 0.19 (0.001 to 0.01) −0.07 (−0.18 to 0.07) 0.165 (0.013 to 0.144)	0.99 0.25 0.009 0.35 0.019	−0.04 (−0.001 to 0.001) −0.09 (−0.24 to 0.11) −0.13 (−0.009 to 0.003) −0.19 (−0.26 to 0.05)	0.76 0.49 0.35 0.17	0.04 (0.00 to 0.001) −0.24 (−0.19 to 0.01) 0.03 (−0.01 to 0.01) −0.36 (−0.37 to 0.04)	0.78 0.07 0.80 0.01	−0.67 (−0.001 to 0.001) 0.16 (−0.03 to 0.10) 0.34 (0.004 to 0.03) 0.04 (−0.29 to 0.39)	0.66 0.27 0.006 0.78

## Data Availability

The data presented in this study are available on reasonable request from the corresponding author. The data are not publicly available due to ethical requirements.

## Abbreviations

CKDu	Chronic Kidney Disease of unknown etiology
KIM-1	Kidney Injury Molecule-1
SCr	Serum Creatinine
eGFR	Estimated Glomerular Filtration Rate
ACR	Albumin Creatinine Ratio
NGAL	Neutrophil Gelatinase-Associated Lipocalin
CINAC	Chronic Interstitial Nephritis in Agricultural Communities
MEN	Meso American Nephropathy
CKD-EPI	CKD-epidemiology collaboration equation
ELISA	Enzyme-Linked Immunosorbent Assay
HPLC	High-Performance Liquid Chromatography

## References

[1] Gianessi L.P. (2013). The increasing importance of herbicides in worldwide crop production. Pest Manag. Sci..

[2] Palis F.G. (2010). Research to Impact: Case Studies for Natural Resource Management for Irrigated Rice in Asia.

[3] Zobiole L.H.S., de Oliveira R.S., Huber D.M., Constantin J., de Castro C., de Oliveira F.A., de Oliveira A. (2010). Glyphosate reduces shoot concentrations of mineral nutrients in glyphosate-resistant soybeans. Plant Soil.

[4] Solomon K.R. (2020). Estimated exposure to glyphosate in humans via environmental, occupational, and dietary pathways: An updated review of the scientific literature. Pest Manag. Sci..

[5] Gasnier C., Dumont C., Benachour N., Clair E., Chagnon M.-C., Séralini G.-E. (2009). Glyphosate-based herbicides are toxic and endocrine disruptors in human cell lines. Toxicology.

[6] Kroncke A.P., Willard M., Huckabee H. (2016). Assessment of Autism Spectrum Disorder: Critical Issues in Clinical, Forensic and School Settings.

[7] Peper E. (2015). Food for thought: Are Herbicides a Factor for the Increase in Allergies and Autism?. Neuroregulation.

[8] Seneff S., Samsel A. (2015). Glyphosate, pathways to modern diseases III: Manganese, neurological diseases, and associated pathologies. Surg. Neurol. Int..

[9] Tsai W.-T. (2013). A review on environmental exposure and health risks of herbicide paraquat. Toxicol. Environ. Chem..

[10] Hoffer E., Taitelman U. (1989). Exposure to Paraquat Through Skin Absorption: Clinical and Laboratory Observations of Accidental Splashing on Healthy Skin of Agricultural Workers. Hum. Toxicol..

[11] Howard J.K. (1980). Paraquat: A Review of Worker Exposure in Normal Usage*. Occup. Med..

[12] Lee K., Park E.-K., Stoecklin-Marois M., Koivunen M.E., Gee S.J., Hammock B.D., Beckett L.A., Schenker M.B. (2009). Occupational paraquat exposure of agricultural workers in large Costa Rican farms. Int. Arch. Occup. Environ. Health.

[13] Mamane A., Baldi I., Tessier J.-F., Raherison C., Bouvier G. (2015). Occupational exposure to pesticides and respiratory health. Eur. Respir. Rev..

[14] Saravu K., Sekhar S., Pai A., Barkur A.S., Rajesh V., Earla J.R. (2013). Paraquat—A deadly poison: Report of a case and review. Indian J. Crit. Care Med..

[15] McKeag D., Maini R., Taylor H.R. (2002). The ocular surface toxicity of paraquat. Br. J. Ophthalmol..

[16] Smith J. (1988). Paraquat Poisoning by Skin Absorption: A Review. Hum. Toxicol..

[17] Tungsanga K., Chusilp S., Israsena S., Sitprija V. (1983). Paraquat poisoning: Evidence of systemic toxicity after dermal exposure. Postgrad. Med. J..

[18] Zhou Y., Vaidya V.S., Brown R.P., Zhang J., Rosenzweig B.A., Thompson K.L., Miller T.J., Bonventre J.V., Goering P.L. (2007). Comparison of Kidney Injury Molecule-1 and Other Nephrotoxicity Biomarkers in Urine and Kidney Following Acute Exposure to Gentamicin, Mercury, and Chromium. Toxicol. Sci..

[19] Suntres E.Z. (2002). Role of antioxidants in paraquat toxicity. Toxicology.

[20] Jayasumana C., Orantes C., Herrera R., Almaguer M., Lopez L., Silva L.C., Ordunez P., Siribaddana S., Gunatilake S., de Broe M.E. (2017). Chronic interstitial nephritis in agricultural communities: A worldwide epidemic with social, occupational and environmental determinants. Nephrol. Dial. Transplant..

[21] Athuraliya N.T.C., Abeysekera T.D.J., Amerasinghe P.H., Kumarasiri R., Bandara P., Karunaratne U., Milton A.H., Jones A.L. (2011). Uncertain etiologies of proteinuric-chronic kidney disease in rural Sri Lanka. Kidney Int..

[22] Wanigasuriya K. (2014). Update on uncertain etiology of chronic kidney disease in Sri Lanka’s north-central dry zone. MEDICC Rev..

[23] Gifford F.J., Gifford R.M., Eddleston M., Dhaun N. (2017). Endemic Nephropathy Around the World. Kidney Int. Rep..

[24] Torres C., Aragón A., González M., López I., Jakobsson K., Elinder C.-G., Lundberg I., Wesseling C. (2010). Decreased Kidney Function of Unknown Cause in Nicaragua: A Community-Based Survey. Am. J. Kidney Dis..

[25] Lunyera J., Mohottige D., von Isenburg M., Jeuland M., Patel U.D., Stanifer J.W. (2016). CKD of Uncertain Etiology: A Systematic Review. Clin. J. Am. Soc. Nephrol..

[26] Babich R., Ulrich J.C., Ekanayake E.D.V., Massarsky A., de Silva P.M.C., Manage P.M., Jackson B.P., Ferguson P.L., di Giulio R.T., Drummond I.A. (2020). Kidney developmental effects of metal-herbicide mixtures: Implications for chronic kidney disease of unknown etiology. Environ. Int..

[27] Jayasumana C., Gunatilake S., Siribaddana S. (2015). Simultaneous exposure to multiple heavy metals and glyphosate may contribute to Sri Lankan agricultural nephropathy. BMC Nephrol..

[28] García-Trabanino R., Jarquín E., Wesseling C., Johnson R.J., González-Quiroz M., Weiss I., Glaser J., Vindell J.J., Stockfelt L., Roncal C. (2015). Heat stress, dehydration, and kidney function in sugarcane cutters in El Salvador—A cross-shift study of workers at risk of Mesoamerican nephropathy. Environ. Res..

[29] Roncaljimenez A.C., García-Trabanino R., Barregard L., Lanaspa M.A., Wesseling C., Harra T., Aragón A., Grases F., Jarquin E.R., González M.A. (2016). Heat Stress Nephropathy From Exercise-Induced Uric Acid Crystalluria: A Perspective on Mesoamerican Nephropathy. Am. J. Kidney Dis..

[30] Jimenez C.A.R., Ishimoto T., Lanaspa M.A., Rivard C.J., Nakagawa T., Ejaz A.A., Cicerchi C., Inaba S., Le M., Miyazaki M. (2014). Fructokinase activity mediates dehydration-induced renal injury. Kidney Int..

[31] Santos U.P., Zanetta D.M.T., Terra-Filho M., Burdmann E.A. (2015). Burnt sugarcane harvesting is associated with acute renal dysfunction. Kidney Int..

[32] Xing X., Lu J., Wang Z. (2015). Associated Risk Factors for Chronic Kidney Disease of Unknown Etiologies in 241 Patients. Int. J. Artif. Organs.

[33] Yang H.-Y., Hung C.-C., Liu S.-H., Guo Y.-G., Chen Y.-C., Ko Y.-C., Huang C.-T., Chou L.-F., Tian Y.-C., Chang M.-Y. (2015). Overlooked Risk for Chronic Kidney Disease after Leptospiral Infection: A Population-Based Survey and Epidemiological Cohort Evidence. PLoS Negl. Trop. Dis..

[34] Nanayakkara S., Senevirathna S.T.M.L.D., Parahitiyawa N.B., Abeysekera T., Chandrajith R., Ratnatunga N., Hitomi T., Kobayashi H., Harada K.H., Koizumi A. (2015). Whole-exome sequencing reveals genetic variants associated with chronic kidney disease characterized by tubulointerstitial damages in North Central Region, Sri Lanka. Environ. Health Prev. Med..

[35] Correa-Rotter R., Wesseling C., Johnson R.J. (2014). CKD of Unknown Origin in Central America: The Case for a Mesoamerican Nephropathy. Am. J. Kidney Dis..

[36] Nerbass F.B., Pecoits-Filho R., Clark W.F., Sontrop J.M., McIntyre C.W., Moist L. (2017). Occupational Heat Stress and Kidney Health: From Farms to Factories. Kidney Int. Rep..

[37] Glaser J., Lemery J., Rajagopalan B., Diaz H.F., García-Trabanino R., Taduri G., Madero M., Amarasinghe M., Abraham G., Anutrakulchai S. (2016). Climate Change and the Emergent Epidemic of CKD from Heat Stress in Rural Communities: The Case for Heat Stress Nephropathy. Clin. J. Am. Soc. Nephrol..

[38] Wesseling C., Aragón A., González M., Weiss I., Glaser J., Bobadilla N.A., Roncal-Jiménez C., Correa-Rotter R., Johnson R.J., Barregard L. (2016). Kidney function in sugarcane cutters in Nicaragua—A longitudinal study of workers at risk of Mesoamerican nephropathy. Environ. Res..

[39] Herath C., Jayasumana C., de Silva P.M.C., de Silva P.C., Siribaddana S., de Broe M.E. (2018). Kidney Diseases in Agricultural Communities: A Case Against Heat-Stress Nephropathy. Kidney Int. Rep..

[40] Jayasumana C., Paranagama P., Agampodi S.B., Wijewardane C., Gunatilake S., Siribaddana S. (2015). Drinking well water and occupational exposure to Herbicides is associated with chronic kidney disease, in Padavi-Sripura, Sri Lanka. Environ. Health.

[41] Jayatilake N., Mendis S., Maheepala P., Mehta F.R. (2013). Chronic kidney disease of uncertain aetiology: Prevalence and causative factors in a developing country. BMC Nephrol..

[42] Wasung M.E., Chawla L.S., Madero M. (2015). Biomarkers of renal function, which and when?. Clin. Chim. Acta.

[43] Waikar S.S., Betensky R.A., Bonventre J.V. (2009). Creatinine as the gold standard for kidney injury biomarker studies?. Nephrol. Dial. Transplant..

[44] Bosch J.P., Saccaggi A., Lauer A., Ronco C., Belledonne M., Glabman S. (1983). Renal functional reserve in humans. Am. J. Med..

[45] Ichimura T., Bonventre J.V., Bailly V., Wei H., Hession C.A., Cate R.L., Sanicola M. (1998). Kidney Injury Molecule-1 (KIM-1), a Putative Epithelial Cell Adhesion Molecule Containing a Novel Immunoglobulin Domain, Is Up-regulated in Renal Cells after Injury. J. Biol. Chem..

[46] Mishra J., Ma Q., Prada A., Mitsnefes M., Zahedi K., Yang J., Barasch J., Devarajan P. (2003). Identification of Neutrophil Gelatinase-Associated Lipocalin as a Novel Early Urinary Biomarker for Ischemic Renal Injury. J. Am. Soc. Nephrol..

[47] Nauta F.L., Boertien W.E., Bakker S.J., van Goor H., van Oeveren W., de Jong P.E., Bilo H., Gansevoort R.T. (2011). Glomerular and Tubular Damage Markers Are Elevated in Patients With Diabetes. Diabetes Care.

[48] Han W.K., Bailly V., Abichandani R., Thadhani R., Bonventre J.V. (2002). Kidney Injury Molecule-1 (KIM-1): A novel biomarker for human renal proximal tubule injury. Kidney Int..

[49] Vaidya V.S., Niewczas M.A., Ficociello L.H., Johnson A.C., Collings F.B., Warram J.H., Krolewski A.S., Bonventre J.V. (2011). Regression of microalbuminuria in type 1 diabetes is associated with lower levels of urinary tubular injury biomarkers, kidney injury molecule-1, and N-acetyl-β-D-glucosaminidase. Kidney Int..

[50] Supavekin S., Zhang W., Kucherlapati R., Kaskel F.J., Moore L.C., Devarajan P. (2003). Differential gene expression following early renal ischemia/reperfusion. Kidney Int..

[51] Yuen P.S.T., Jo S.-K., Holly M.K., Hu X., Star R.A. (2006). Ischemic and nephrotoxic acute renal failure are distinguished by their broad transcriptomic responses. Physiol. Genom..

[52] Argyropoulos C.P., Chen S.S., Ng Y.-H., Roumelioti M.-E., Shaffi K., Singh P.P., Tzamaloukas A.H. (2017). Rediscovering Beta-2 Microglobulin As a Biomarker across the Spectrum of Kidney Diseases. Front. Med..

[53] Nickolas T.L., O’Rourke M.J., Yang J., Sise M.E., Canetta P.A., Barasch N., Buchen C., Khan F., Mori K., Giglio J. (2008). Sensitivity and Specificity of a Single Emergency Department Measurement of Urinary Neutrophil Gelatinase-Associated Lipocalin for Diagnosing Acute Kidney Injury. Ann. Intern. Med..

[54] De Silva P.M.C.S., Abdul K.S.M., Eakanayake E.M.D.V., Jayasinghe S.S., Jayasumana C., Asanthi H.B., Perera H.S.D., Chaminda G.G.T., Chandana E.P.S., Siribaddana S.H. (2016). Urinary Biomarkers KIM-1 and NGAL for Detection of Chronic Kidney Disease of Uncertain Etiology (CKDu) among Agricultural Communities in Sri Lanka. PLoS Neglected Trop. Dis..

[55] Gunasekara T., de Silva P.M.C., Herath C., Siribaddana S., Siribaddana N., Jayasumana C., Jayasinghe S., Cardenas-Gonzalez M., Jayasundara N. (2020). The Utility of Novel Renal Biomarkers in Assessment of Chronic Kidney Disease of Unknown Etiology (CKDu): A Review. Int. J. Environ. Res. Public Health.

[56] Levey A.S., Stevens L.A., Schmid C.H., Zhang Y.L., Castro A.F., Feldman H.I., Kusek J.W., Eggers P., van Lente F., Greene T. (2009). A new equation to estimate glomerular filtration rate. Ann. Intern. Med..

[57] Acquavella J.F., Alexander B.H., Mandel J.S., Gustin C., Baker B., Chapman P., Bleeke M. (2004). Glyphosate biomonitoring for farmers and their families: Results from the Farm Family Exposure Study. Environ. Health Perspect..

[58] Bolognesi C., Carrasquilla G., Volpi S., Solomon K.R., Marshall E.J.P. (2009). Biomonitoring of Genotoxic Risk in Agricultural Workers from Five Colombian Regions: Association to Occupational Exposure to Glyphosate. J. Toxicol. Environ. Health Part A.

[59] Cha E.S., Lee Y.K., Moon E.K., Kim Y.B., Lee Y.-J., Jeong W.C., Cho E.Y., Lee I.J., Hur J., Ha M. (2012). Paraquat application and respiratory health effects among South Korean farmers. Occup. Environ. Med..

[60] Kongtip P., Nankongnab N., Phupancharoensuk R., Palarach C., Sujirarat D., Sangprasert S., Sermsuk M., Sawattrakool N., Woskie S.R. (2017). Glyphosate and Paraquat in Maternal and Fetal Serums in Thai Women. J. Agromed..

[61] Mariager T.P., Madsen P.V., Ebbehøj N.E., Schmidt B., Juhl A. (2013). Severe adverse effects related to dermal exposure to a glyphosate-surfactant herbicide. Clin. Toxicol..

[62] Curwin B.D., Hein M.J., Sanderson W.T., Striley C., Heederik D., Kromhout H., Reynolds S.J., Alavanja M.C. (2006). Urinary Pesticide Concentrations Among Children, Mothers and Fathers Living in Farm and Non-Farm Households in Iowa. Ann. Occup. Hyg..

[63] Schledorn M.K.P. (2014). Detection of Glyphosate Residues in Animals and Humans. J. Environ. Anal. Toxicol..

[64] Niemann L., Sieke C., Pfeil R., Solecki R. (2015). A critical review of glyphosate findings in human urine samples and comparison with the exposure of operators and consumers. J. Consum. Prot. Food Saf..

[65] Baharuddin M.R.B., Sahid I.B., Noor M.A.B.M., Sulaiman N., Othman F. (2011). Pesticide risk assessment: A study on inhalation and dermal exposure to 2,4-D and paraquat among Malaysian paddy farmers. J. Environ. Sci. Health Part B.

[66] Joode B.N.V.W.D., de Graaf I.A.M., Wesseling C., Kromhout H. (1996). Paraquat Exposure of Knapsack Spray Operators on Banana Plantations in Costa Rica. Int. J. Occup. Environ. Health.

[67] Huang Y., He Q. (2017). Inhibition of c-Src protects paraquat induced microvascular endothelial injury by modulating caveolin-1 phosphorylation and caveolae mediated transcellular permeability. Environ. Toxicol. Pharmacol..

[68] Weng C.-H., Hu C.-C., Lin J.-L., Lin-Tan D.-T., Huang W.-H., Hsu C.-W., Yen T.-H. (2012). Sequential Organ Failure Assessment Score Can Predict Mortality in Patients with Paraquat Intoxication. PLoS ONE.

[69] Shi X.-B., He J.-L., Lu Y.-Q. (2013). The significance of urine N-acetyl-beta-D-glucosaminidase in kidney injury with patients acute paraquat poisoning. Zhonghua Laodong Weisheng Zhiyebing Zazhi = Chin. J. Ind. Hyg. Occup. Dis..

[70] Mohamed F., Buckley N.A., Jayamanne S., Pickering J.W., Peake P., Palangasinghe C., Wijerathna T., Ratnayake I., Shihana F., Endre Z.H. (2015). Kidney damage biomarkers detect acute kidney injury but only functional markers predict mortality after paraquat ingestion. Toxicol. Lett..

[71] Mohamed F., Buckley N.A., Pickering J.W., Wunnapuk K., Dissanayake S., Chathuranga U., Gawarammana I., Jayamanne S., Endre Z.H. (2017). Nephrotoxicity-induced proteinuria increases biomarker diagnostic thresholds in acute kidney injury. BMC Nephrol..

[72] Orantes C.M., Herrera R., Almaguer M., Brizuela E.G., Núñez L., Alvarado N.P., Fuentes E.J., Bayarre H.D., Amaya J.C., Calero D.J. (2014). Epidemiology of chronic kidney disease in adults of Salvadoran agricultural communities. MEDICC Rev..

[73] Laws R.L., Brooks D.R., Amador J.J., Weiner D.E., Kaufman J.S., Ramírez-Rubio O., Riefkohl A., Scammell M.K., López-Pilarte D., Sánchez J.M. (2015). Changes in kidney function among Nicaraguan sugarcane workers. Int. J. Occup. Environ. Health.

[74] Charlton J.R., Portilla D., Okusa M.D. (2014). A basic science view of acute kidney injury biomarkers. Nephrol. Dial. Transplant..

[75] Wunnapuk K., Liu X., Peake P., Gobe G., Endre Z., Grice J.E., Roberts M.S., Buckley N.A. (2013). Renal biomarkers predict nephrotoxicity after paraquat. Toxicol. Lett..

[76] Ahn J.Y., Lee M.J., Seo J.S., Choi D., Park J.B. (2016). Plasma neutrophil gelatinase-associated lipocalin as a predictive biomarker for the detection of acute kidney injury in adult poisoning. Clin. Toxicol..

[77] Gil H.-W., Yang J.-O., Lee E.-Y., Hong S.-Y. (2009). Clinical implication of urinary neutrophil gelatinase-associated lipocalin and kidney injury molecule-1 in patients with acute paraquat intoxication. Clin. Toxicol..

[78] Roberts D.M., Wilks M.F., Roberts M.S., Swaminathan R., Mohamed F., Dawson A.H., Buckley N.A. (2011). Changes in the concentrations of creatinine, cystatin C and NGAL in patients with acute paraquat self-poisoning. Toxicol. Lett..

[79] Anand S., Caplin B., Gonzalez-Quiroz M., Schensul S.L., Bhalla V., Parada X., Nanayakkara N., Fire A., Levin A., Friedman D.J. (2019). Epidemiology, molecular, and genetic methodologies to evaluate causes of CKDu around the world: Report of the Working Group from the ISN International Consortium of Collaborators on CKDu. Kidney Int..

[80] Osten J.R.-V., Dzul-Caamal R. (2017). Glyphosate Residues in Groundwater, Drinking Water and Urine of Subsistence Farmers from Intensive Agriculture Localities: A Survey in Hopelchén, Campeche, Mexico. Int. J. Environ. Res. Public Health.

[81] Rubio F., Veldhuis L.J., Clegg B.S., Fleeker J.R., Hall J.C. (2003). Comparison of a Direct ELISA and an HPLC Method for Glyphosate Determinations in Water. J. Agric. Food Chem..

[82] Wanigasuriya K., Jayawardene I., Amarasiriwardena C., Wickremasinghe R. (2017). Novel urinary biomarkers and their association with urinary heavy metals in chronic kidney disease of unknown aetiology in Sri Lanka: A pilot study. Ceylon Med. J..

[83] Cárdenas-González M., Osorio-Yáñez C., Gaspar-Ramírez O., Pavković M., Ochoa-Martínez A., López-Ventura D., Medeiros M., Barbier O., Pérez-Maldonado I., Sabbisetti V. (2016). Environmental exposure to arsenic and chromium in children is associated with kidney injury molecule-1. Environ. Res..

[84] Prozialeck W.C., Edwards J.R. (2010). Early biomarkers of cadmium exposure and nephrotoxicity. BioMetals.

